# Morphological and functional characteristics of the meibomian gland in pediatric patients with epiblepharon

**DOI:** 10.1186/s12886-024-03345-5

**Published:** 2024-02-22

**Authors:** Junping Li, Xiaolin Qin, Hong Jiang, Yunan Guo, Xin Liu, Rui Zhang, Lu Jiang, Anshi Du

**Affiliations:** 1 Department of Plastic Surgery, Aier Eye Hospital (East of Chengdu), No. 388 Shuanglin Road, Chenghua District, 610051 Chengdu, Sichuan Province China; 2 Department of Pediatric Ophthalmology, Aier Eye Hospital (East of Chengdu), 610051 Chengdu, China

**Keywords:** Meibomian gland, Dry eye, MGD, Epiblepharon

## Abstract

**Background:**

To observe morphologic and functional changes in meibomian glands in pediatric patients with and without lower eyelid epiblepharon.

**Methods:**

In this prospective observation study, 55 eyes of 55 patients( 24 males, 31 females; mean age ± SD,9.82 ± 2.59 years; range 6–14 years) and 60 eyes of 60 controls ( 32 males, 28 females; mean age ± SD,10.57 ± 2.75 years; range 6–14 years) were included. The following tests were performed: eyelid margin abnormality by slit-lamp examination, measurement of noninvasive keratographic break-up time (NIKBUT), grading of absence of meibomian gland (meibography score) assessed with noncontact meibography, morphologic changes of meibomian glands (thinning, dilatation and distortion), tear production by the Schirmer 1 test, and grading of meibum quality and meibomian gland expressibility.

**Results:**

The morphologic changes in meibomian glands were more common in the epiblepharon group (56.36%) than in the control group (28.33%) (*p* = 0.002). The meibum quality was worse in the epiblepharon group than in the control group (*p* = 0.009), and the NIKBUT was significantly shorter in the epiblepharon group than in the control group (*p* = 0.012). There was no significant difference in the Schirmer 1 test, meibomian gland expressibility, eyelid margin abnormality score or total meibography score between the two groups. Morphologic changes in the meibomian glands in the upper eyelids (38.18%) were more common than those in the lower eyelids (20%) (*p* = 0.036) in the epiblepharon group, and the meibography score was higher in the upper eyelids than in the lower eyelids (*p* = 0.001).

**Conclusion:**

There are morphological and functional changes in meibomian glands in pediatric patients with lower eyelid epiblepharon. Although the inverted eyelashes were located in the lower eyelid, morphological changes in the meibomian glands were more common in the upper eyelid.

## Background

Epiblepharon is a common eye disease in Asian children, with an incidence of 46-52.5% [1]. It involves a horizontal skin fold overlapping the lid margin, resulting in the eyelashes brushing against the ocular surface, and the lower eyelids are much more commonly affected [2].Children often have clinical manifestations such as blinking, photophobia, and eye redness.

In recent years, with the increase in research on dry eye in children and meibomian gland dysfunction (MGD), it has been found that a variety of diseases may be related to dry eye in children and MGD, such as allergic conjunctivitis, blepharitis and refractive error [3–5]. However, it is unknown whether epiblepharon is related to dry eye in children and MGD. Previous studies have shown that in elderly patients with involutional entropion, in addition to the symptoms caused by mechanical stimulation, it also affected the structure and function of the meibomian gland and even led to MGD [6]. Epiblepharon is also a disease with abnormal eyelid position, but it is unknown whether epiblepharon has an effect on the morphology and function of meibomian glands. However, abnormalities in the morphology and function of meibomian glands are a contributing cause of evaporative dry eye [7, 8]. Therefore, the purpose of this study was to understand the morphological and functional characteristics of meibomian glands in pediatric patients with epiblepharon.

## Subjects and methods

### Subjects

From June 2019 to March 2022, 55 eyes of 55 patients with lower eyelid epiblepharon (24 males and 31 females; mean age ± SD, 9.82 ± 2.59 years, range 6–14 years). These 55 patients were all intended for epiblepharon correction, the range of trichiasis was >1/3,<1/2 eyelid length. As a control group, 60 eyes of 60 children (32 males and 28 females; mean age ± SD, 10.57 ± 2.75 years, range 6–14 years) were included. Exclusion criteria for the two groups included blepharitis, contact lens wear, obvious eyelid or ocular surface disorders, atopic dermatitis, continuous eyedrop use, a history of eye surgery, systemic or ocular diseases that would interfere with tear film production or function, and children who could not cooperate. Data used were obtained from the left eye of each subject except when the epiblepharon was unilateral. If the left eye was excluded from the study, data from the right eye were used. Written informed consent was obtained from the patient’s parents before examination. This study was approved by the Ethics Committee of Aier Eye Hospital (East of Chengdu) and followed the tenets of the Declaration of Helsinki.

### Examinations

The assessment was performed sequentially as follows. First slit-lamp examination of eyelid margin abnormalities was performed. The eyelid margin was evaluated with scores from 0 to 4 according to irregular lid margin, vascular engorgement, meibomian gland opening obstruction, and anterior or posterior replacement of the mucocutaneous junction. Noninvasive keratographic break-up time (NIKBUT) was captured by a noncontact meibography system (Kerotography 5 M, Oculus, Wetzlar, Germany), and the average time was used. Infrared images of the meibomian glands were captured after the eyelids were everted by Keratography 5 M. Partial or complete loss of the meibomian glands was scored using the following grades (meibography score) for the upper and lower eyelids separately: grade 0 (no loss of meibomian glands), grade 1 (loss of less than one-third of the total area of meibomian glands), grade 2 (loss of between one-third and two-thirds of the total area), and grade 3 (loss of over two-thirds of the total area), and the total score was 0–6 for each eye [9–10]. Meanwhile, the presence of morphological changes in the upper eyelid and lower eyelid were evaluated. Thinning, dilatation, distortion (>45º) of at least one meibomian duct in the upper or lower eyelid was defined as morphologic change, the presence of thinning and dilatation of meibomian gland ducts were determined when there was a relative decrement and increment in duct diameter [11–12].The upper and lower lids of the same eye were not counted repeatedly when the two groups were compared. Tear film production was evaluated by the Schirmer 1 test without applying topical anesthetics. Taking into account the child’s cooperation, examinations of meibum quality and meibomian gland expressibility were performed with meibomian forceps under topical anesthesia while the child was lying down or under general anesthesia during the operation. During the examination, it was found that the temporal side was easier to cooperate in some children, this may be related to the fact that trichiasis was mainly located on the nasal side, and the nasal cornea was more likely to be involved. For consistency, five meibomian glands in the temporal lobe were selected as evaluation objects, rather than examining 3 locations (nasal, medial, and temporal), and scores were assigned based on the International MGD Working Group Standard: 0, all five glands have secretion discharge capacity; 1, three or four glands have secretion discharge capacity; 2, one or two glands have secretion discharge capacity; and 3, no glands have secretion discharge capacity. Meibum quality was assessed on a scale of 0 to 3: 0, clear; 1, cloudy; 2, cloudy with debris (granular); and 3, thick, similar to toothpaste [9–10]. All examinations were conducted by the same doctor during the whole course of the study.

### Statistical analyses

Statistical analysis was performed using SPSS 25.0. Continuous variables between both groups were tested with an independent t test, binary variables were tested with the chi-square test, and categorical variables were tested with a nonparametric test. Independent samples t tests, Mann-Whitney U tests and chi-square tests were used to compare variables between the groups. The statistical significance level was set at a P value of < 0.05.

## Results

**Fig. 1:**
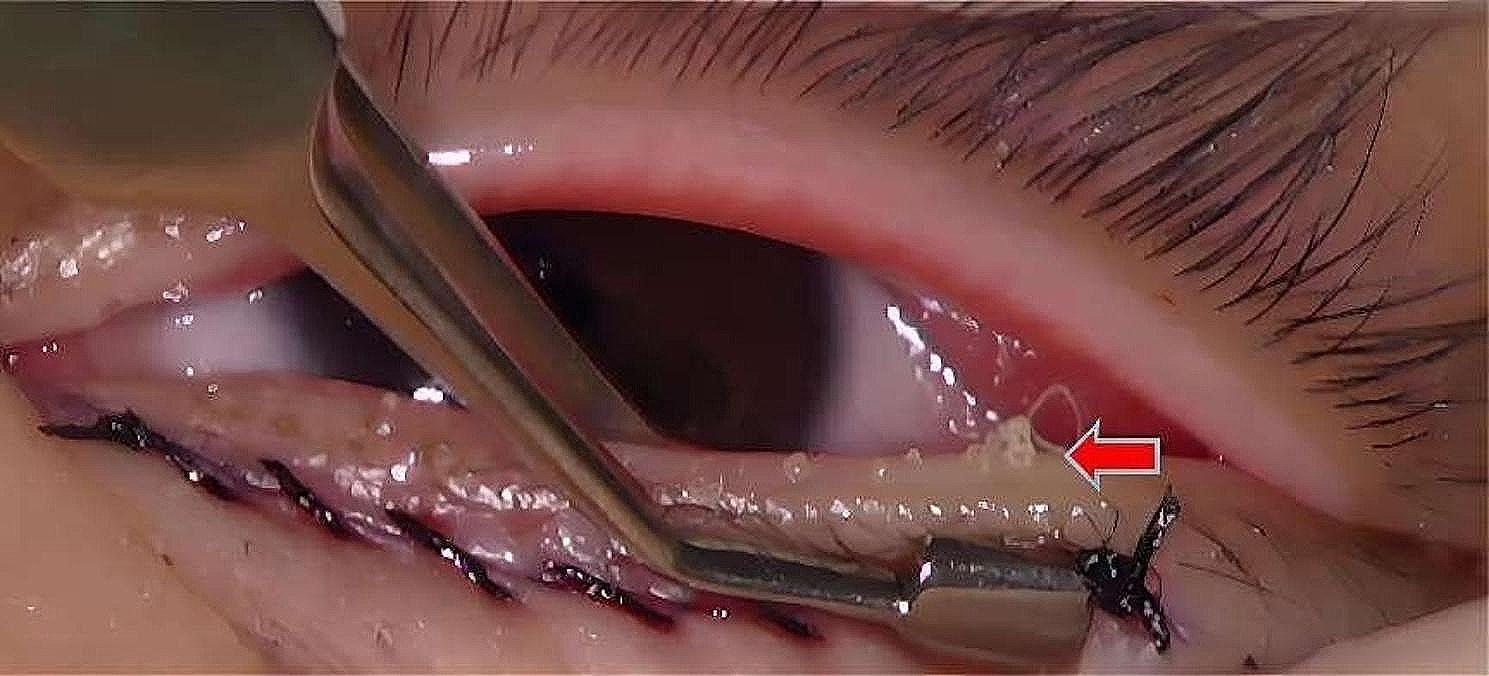
Examinations of meibum quality under general anesthesia after the operation. The meibum were similar to toothpaste (red arrow)

**Fig. 2:**
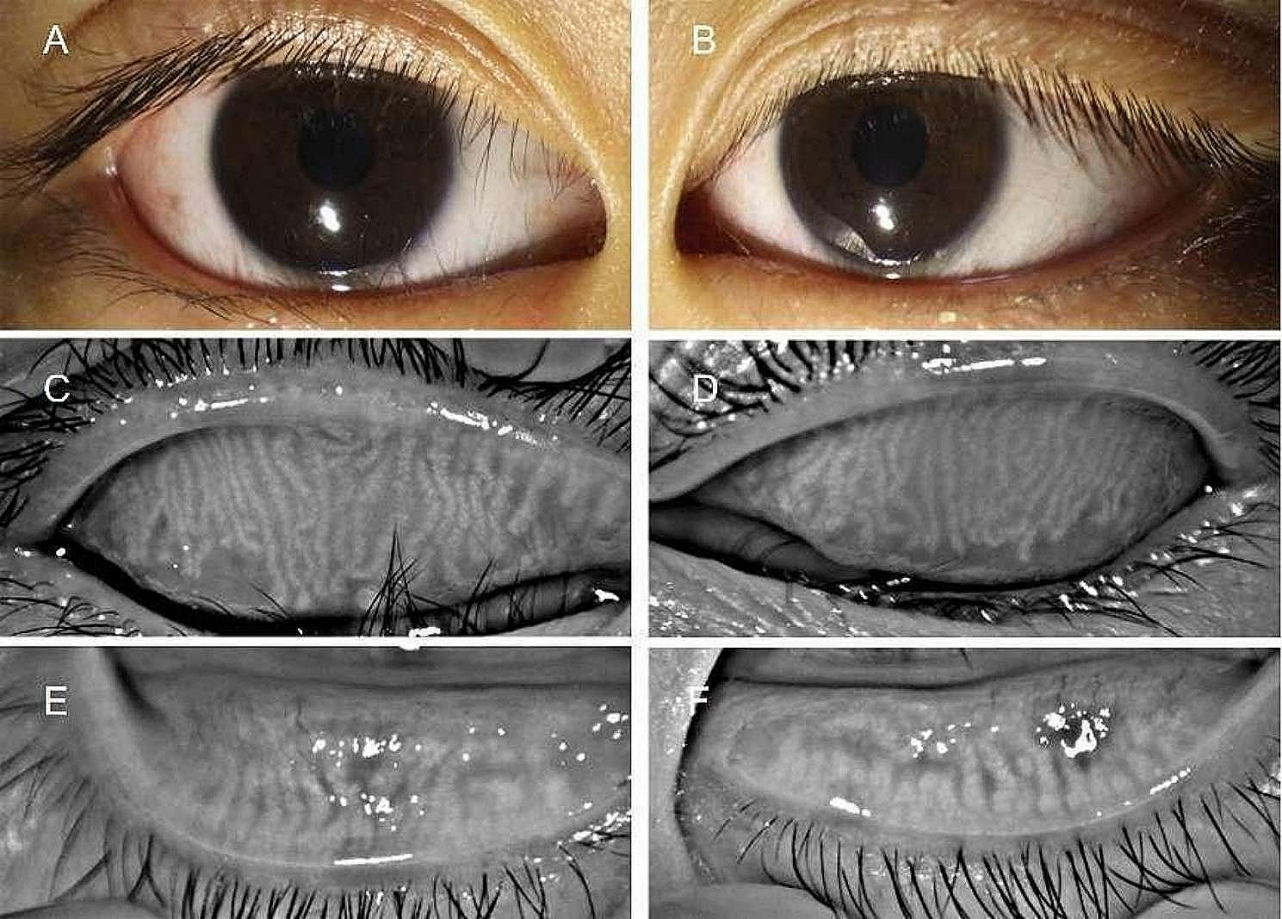
The morphological changes in meibomian glands in the epiblepharon patient. (**A**) and (**B**) show the eyelashes on the nasal side of the lower eyelid touch the eyeball. (**C**) and (**D**) show the morphological changes (thinning, dilatation and distortion (> 45º) in meibomian glands in the upper eyelid, less than one-third of the meibomian gland area was absence. E and F show a small meibomian gland area on the inside was absence)

Table 1 shows the results of examinations between the epiblepharon and control groups. The morphological changes in meibomian glands (thinning, dilatation and distortion (> 45º) of the ducts) were observed in 31 of 55 eyes (56.36%) in the epiblepharon group and 17 of 60 eyes (28.33%) in the control group (*p* = 0.002). The meibum quality was worse in the epiblepharon group than in the control group (*p* = 0.009), and severe cases were similar to toothpaste (Fig. 1). The NIKBUT was significantly shorter in the epiblepharon group than in the control group (*p* = 0.012). There was no significant difference in the Schirmer 1 test, meibomian gland expressibility, eyelid margin abnormality score or total meibomian gland absence score between the two groups. Table 2 shows the comparison of the results between the upper and lower eyelids in the epiblepharon group. The morphological changes in meibomian glands were observed in 21 of 55 eyelids (38.1%) in the upper eyelids and 11 of 55 eyelids (20%) in the lower eyelids (*p* = 0.036) (Fig. 2). The meibomian gland absence score was higher in the upper eyelids than in the lower eyelids (*p* = 0.001). There was no significant difference in meibomian gland expressibility and meibum quality between the upper and lower eyelids. Table 3 shows the comparison of the results between the upper and lower eyelids in the control group, there was no significant difference between the upper and lower eyelids.

**Table 1:** Comparison of morphological and functional indexes between the two groups

	Epiblepharon(*n* = 55)	Control (*n* = 60)	t/Z	p
Gender (Male/Female)	24/31	32/28	1.080	0.299
Age (mean ± SD), yr	9.82 ± 2.59(6–14)^‡^	10.57 ± 2.75(6–14)^‡^	-1.499	0.137
NIKBUT(mean ± SD), s	8.49 ± 5.64(2.29–23.31)^‡^	11.09 ± 5.25(2.17–24.03)^‡^	-2.555	0.012*
Schirmer test(mean ± SD), mm	15.07 ± 3.46(8–23)^‡^	15.17 ± 3.83(6–24)^‡^	-0.138	0.891
Meibomian gland expressibility	0(0,1)^†^(0–3)^‡^	0(0,0.75)^†^(0–3)^‡^	-0.87	0.38
Meibum quality	1(0,2)^†^(0–6)^‡^	0(0,1)^†^(0–6)^‡^	-2.615	0.009*
Eyelid margin abnormality score	0(0,1)^†^(0–2)^‡^	0(0,0.75)^†^(0–2)^‡^	-1.464	0.143
Total meibomian gland absence score(meibography scores)	0(0,1)^†^(0–6)^‡^	0(0,1)^†^(0–2)^‡^	-1.192	0.233
Morphological changes in Meibomian glands (n, %)	31(56.36%)	17(28.33%)	9.272	0.002*

NIKBUT: noninvasive keratographic break-up time

^†^ quartile range

^‡^the range of the corresponding data

**Table 2:** Comparison of morphological and functional indexes of upper and lower in epiblepharon group

	Upper eyelid	Lower eyelid	Z	p
Morphologic changes of meibomian glands (n, %)	21(38.18%)	11(20%)	4.407	0.036*
Meibomian gland expressibility	0(0,0)^†^(0–2)^‡^	0(0,0)^†^(0–2)^‡^	-0.053	0.958
Meibum quality	1(0,1)^†^(0–3)^‡^	1(0,1)^†^(0–3)^‡^	-0.198	0.843
Meibomian gland absence score(meibography scores)	0(0,1)^†^(0–3)^‡^	0(0,0)^†^(0–3)^‡^	-3.238	0.001*

^†^ quartile range

^‡^the range of the corresponding data

**Table 3:** Comparison of morphological and functional indexes of upper and lower in control group

	Upper eyelid	Lower eyelid	Z	p
Morphologic changes of meibomian glands (n, %)	15(25%)	7(11.67%)	3.562	0.059
Meibomian gland expressibility	0(0,1)^†^(0–1)^‡^	0(0,1)^†^(0–2)^‡^	-1.546	0.122
Meibum quality	0(0,3)^†^(0–3)^‡^	0(0,1)^†^(0–3)^‡^	-0.057	0.954
Meibomian gland absence score(meibography scores)	0(0,1)^†^(0–1)^‡^	0(0,1)^†^(0–1)^‡^	-1.680	0.093

^†^ quartile range, ^‡^the range of the corresponding data

## Discussion

The findings from this study demonstrate that morphological abnormalities of meibomian glands (thinning, dilatation and distortion (> 45º) of the ducts) are significantly more common in patients with epiblepharon than in those without epiblepharon, and there is an interesting phenomenon: the abnormalities are more common in the upper eyelid, although epiblepharon occurs in the lower eyelid. The exact mechanisms are unclear, and there are two possible reasons based on previous studies: on the one hand, patients with epiblepharon frequently rub their eyes because of discomfort, and rubbing has been reported to induce keratoconus [13–15]. When the eye is rubbed, the mechanical force produced is directly applied to the eyelid,therefore, rubbing might also induce the morphological abnormality of meibomian gland ducts, which is also observed in patients with allergic conjunctivitis [16]. The pressure is mainly concentrated on the upper eyelid when robbing eyes, so the meibomian gland ducts in the upper eyelid are more likely to be affected. The presence of eye rubbing in each subject was not investigated; therefore, the association between eye rubbing and the morphological abnormalities of meibomian glands was not clarified in this study. Further study was necessary to elucidate this point. On the other hand, chronic inflammation because of persistent mechanical trauma by eyelashes might also be a reason [17]. It was reported that inflammatory disorders of the ocular surface may affect the structure and function of the meibomian gland [11, 18–19]. When inflammatory cells infiltrated the meibomian glands, normal meibum secretion was blocked, but abnormal meibum continued to be produced, and these changes led to increased pressure in the glands, which may cause the morphological abnormalities of meibomian gland [20–21]. At the same time, inflammation may accelerate acinar atrophy [22]. The meibomian gland ducts in the upper eyelid are longer than those in the lower eyelid, and the meibum is more difficult to secrete and more prone to morphological abnormalities.

The meibum quality may prioritize the inflammatory stimulus [20]. As we observed, the meibum score was higher in the epiblepharon group than in the control group, there was even toothpaste-like meibum in some patients. In this study, although the nasal side of meibomian glands was not examined directly because of the children’s lack of cooperation. According to our observations and previous literature [18–19], the ocular surface inflammation caused by epiblepharon involved all meibomian glands, not just the nasal side. From another perspective, if the temporal side function was already affected, then the nasal side function was more likely to change which was the location of the epiblepharon. Previous studies have shown that meibomian gland expressibility is decreased in patients with involutional entropion [6]. However, there were no significant differences in meibomian gland expressibility between the two groups in this study. Two reasons could account for this result. First, meibomian gland expressibility is related not only to meibum quality but also to whether the meibomian gland orifice is blocked. Although both involutional entropion and epiblepharon can cause ocular surface inflammation, epiblepharon is different from involutional entropion in anatomy, its eyelid margin and meibomian gland orifice were not soaked in tears [2, 6], inflammation caused by epiblepharon had little effect on the lid margin based on our observations, and the meibomian gland orifice was basically normal. Therefore, the meibum can be discharged smoothly. Second, although there were obvious morphological abnormalities of meibomian gland, there was no significant difference in meibomian gland absence compared with the control group. It can be understood that the above morphological abnormalities are not enough to affect the meibomian gland secretion function [23]. Combined with the above factors, meibomian gland expressibility may not be affected.

The NIKBUT in the epiblepharon group was shorter than that in the control group. This may be related to the decreased tear film stability caused by mechanical stimulation of the eyelashes. However, there is one thing that needs attention: the intact corneal epithelium was destroyed due to eyelash friction, which may have implications for NIKBUT results.

Another interesting phenomenon in this study was that meibomian gland absence in the upper eyelid was more common than in the lower eyelid in the epiblepharon group, although there were no significant differences in the total meibomian gland absence score between the two groups. This is consistent with previous studies. Several studies have reported that a certain percentage of meibomian gland absence is present in normal asymptomatic children [12, 24]. The meibomian gland in the upper eyelid is long, and the meibum quality is more difficult to secrete, which may be the main reasons why meibomian gland absence is more common in the upper eyelid.

A limitation of this study is that the assessment of meibomian gland expressibility was performed with a meibomian gland massage clip under anesthesia, which may differ from that with meibomian gland evaluation. However, these operations were performed by the same doctor with the same massage clips in the two groups. The second limitation of the study is that the doctor cannot be blinded when assessing results. Another limitation of the study is that we assessed the meibomian glands at only one point in time; if there were a comparison before and after surgery, this study would be more complete. This will be the direction of our further research.

## Conclusion

Morphological and functional abnormalities of the meibomian glands are more common in pediatric patients with epiblepharon. These children may develop MGD or dry eye. While treating epiblepharon, it is also necessary to pay attention to the structure and functional status of their meibomian glands. If the morphology or function of the meibomian gland has changed, appropriate treatment was required. With adequate screening, patients with structural and functional abnormalities of the meibomian glands can be identified and treated promptly in the disease course, with likely improved long-term outcomes. Whether the morphology and function of meibomian glands will improve after epiblepharon correction needs further study.

## Data Availability

The datasets used and/or analyzed during the current study are available from the corresponding author on reasonable request.

## Abbreviations

MGD: Meibomian gland dysfunction
NIKBUT: Noninvasive keratographic break-up time
